# Cardiovascular magnetic resonance assessment of acute cardiovascular effects of voluntary apnoea in elite divers

**DOI:** 10.1186/s12968-018-0455-x

**Published:** 2018-06-18

**Authors:** L. Eichhorn, J. Doerner, J. A. Luetkens, J. M. Lunkenheimer, R. C. Dolscheid-Pommerich, F. Erdfelder, R. Fimmers, J. Nadal, B. Stoffel-Wagner, H. H. Schild, A. Hoeft, B. Zur, C. P. Naehle

**Affiliations:** 1 Department of Anaesthesiology and Intensive Care Medicine, University Hospital of Bonn, Bonn, Germany; 2 Department of Radiology, University Hospital of Bonn, Bonn, Germany; 3 Department of Radiology, University Hospital of Cologne, Cologne, Germany; 4 Institute for Medical Biometry, Informatics and Epidemiology (IMBIE), Bonn, Germany; 5 Medical Biometry, Information Technology and Epidemiology, University of Bonn, Bonn, Germany

**Keywords:** Hypoxia, Apnoea, Cardiac function, CMR, Catecholamine, hs-cT

## Abstract

**Background:**

Prolonged breath holding results in hypoxemia and hypercapnia. Compensatory mechanisms help maintain adequate oxygen supply to hypoxia sensitive organs, but burden the cardiovascular system.

The aim was to investigate human compensatory mechanisms and their effects on the cardiovascular system with regard to cardiac function and morphology, blood flow redistribution, serum biomarkers of the adrenergic system and myocardial injury markers following prolonged apnoea.

**Methods:**

Seventeen elite apnoea divers performed maximal breath-hold during cardiovascular magnetic resonance imaging (CMR). Two breath-hold sessions were performed to assess (1) cardiac function, myocardial tissue properties and (2) blood flow. In between CMR sessions, a head MRI was performed for the assessment of signs of silent brain ischemia. Urine and blood samples were analysed prior to and up to 4 h after the first breath-hold.

**Results:**

Mean breath-hold time was 297 ± 52 s. Left ventricular (LV) end-systolic, end-diastolic, and stroke volume increased significantly (*p* < 0.05). Peripheral oxygen saturation, LV ejection fraction, LV fractional shortening, and heart rate decreased significantly (*p* < 0.05). Blood distribution was diverted to cerebral regions with no significant changes in the descending aorta. Catecholamine levels, high-sensitivity cardiac troponin, and NT-pro-BNP levels increased significantly, but did not reach pathological levels.

**Conclusion:**

Compensatory effects of prolonged apnoea substantially burden the cardiovascular system. CMR tissue characterisation did not reveal acute myocardial injury, indicating that the resulting cardiovascular stress does not exceed compensatory physiological limits in healthy subjects. However, these compensatory mechanisms could overly tax those limits in subjects with pre-existing cardiac disease. For divers interested in competetive apnoea diving, a comprehensive medical exam with a special focus on the cardiovascular system may be warranted.

**Trial registration:**

This prospective single-centre study was approved by the institutional ethics committee review board. It was retrospectively registered under ClinicalTrials.gov (Trial registration: NCT02280226. Registered 29 October 2014).

**Electronic supplementary material:**

The online version of this article (10.1186/s12968-018-0455-x) contains supplementary material, which is available to authorized users.

## Background

Hypoxia is associated with significant changes to the cardiovascular system. It is known from animal studies that hypoxia is associated with increased mean arterial blood pressure and altered myocardial morphometry due to increased left ventricular (LV) end-diastolic pressure with lengthening of end-diastolic and end-systolic myocardial fibres [1, 2]. In humans, prolonged breath-hold – also termed [voluntary] apnoea – can be used for studying the cardiovascular adaptations to acute dynamic hypoxemia and hypercapnia [3, 4]. Trained apnoea divers are able to achieve breath-hold durations of more than 6 minutes on a regular basis. Apnoea itself leads to hypoxia and hypercapnia, both in turn leading to an activation of the sympathetic nervous system and ultimately causing peripheral vasoconstriction [5]. At the end of apnoea, trained breath-hold divers can achieve hypoxic states with end tidal pO_2_ levels of < 30 mmHg O_2_ [6, 7]. Adequate oxygen supply of hypoxia sensitive organs (e. g. the brain) is assured by the so-called diving response, which initiates a preferential redistribution of blood flow to the brain and the heart [3, 8]. The diving response comprises bradycardia and peripheral vasoconstriction, with the latter causing severe hypertension. In some cases cardiac complications such as cardiac arrhythmias were observed [8, 9], possibly due to a transient, but marked LV dilation during prolonged apnoea [10]. Whether voluntary apnoea with its cardiovascular burden leads to measurable changes of cardiac biomarkers, especially those with high sensitivity, as a precursor of cardiomyocyte injury has not been evaluated yet.

Cardiovascular magnetic resonance (CMR) is a standard non-invasive method for functional analysis of the heart [11], which allows for a high-resolution, three-dimensional anatomical and functional visualization of the heart. Furthermore, CMR facilitates quantitative assessment of blood flow in the vascular system and can therefore determine fast and repetitive blood flow distribution under apnoea.

The aim of this study was to investigate cardiovascular effects during maximal apnoea in elite healthy subjects and to determine the accompanied blood flow redistribution. Cardiac biomarkers and serum markers of the adrenergic system were determined. Cerebral MRI was performed after maximal apnoea to detect potential brain injury.

## Methods

### Inclusion and exclusion criteria

Inclusion criteria were experience in apnoea diving with a minimum breath-hold time of 270 s, a minimum age of 18 years, an unremarkable history of cardiac and lung disease, and the absence of long-term medication. Exclusion criteria were any contraindication to CMR or any known heart or lung disease. Participants were required not to drink caffeine-containing drinks and were instructed not to eat at least 8 h before the examination.

All study subjects received an information sheet 14 days prior to the study. Informed consent was obtained from all participants prior to study inclusion. Participants were questioned about training protocols and diving experience.

### Study protocol

The study protocol comprised two apnoea sessions of individual maximum breath-hold combined with CMR measurements (one “functional cardiac session” and one “flow session”). Participants were asked to perform their usual pre-apnoea routines (yoga and breathing exercises). Fifteen minutes before CMR the participants were required to stop with their individual exercises and to breathe normally. A maximum of three deep inspirations prior to the final breath-hold was allowed. Hyperventilation was not allowed. Apnoea was performed as long as the individual subjects were able to withstand the breathing reflex.

**Fig. 1:**
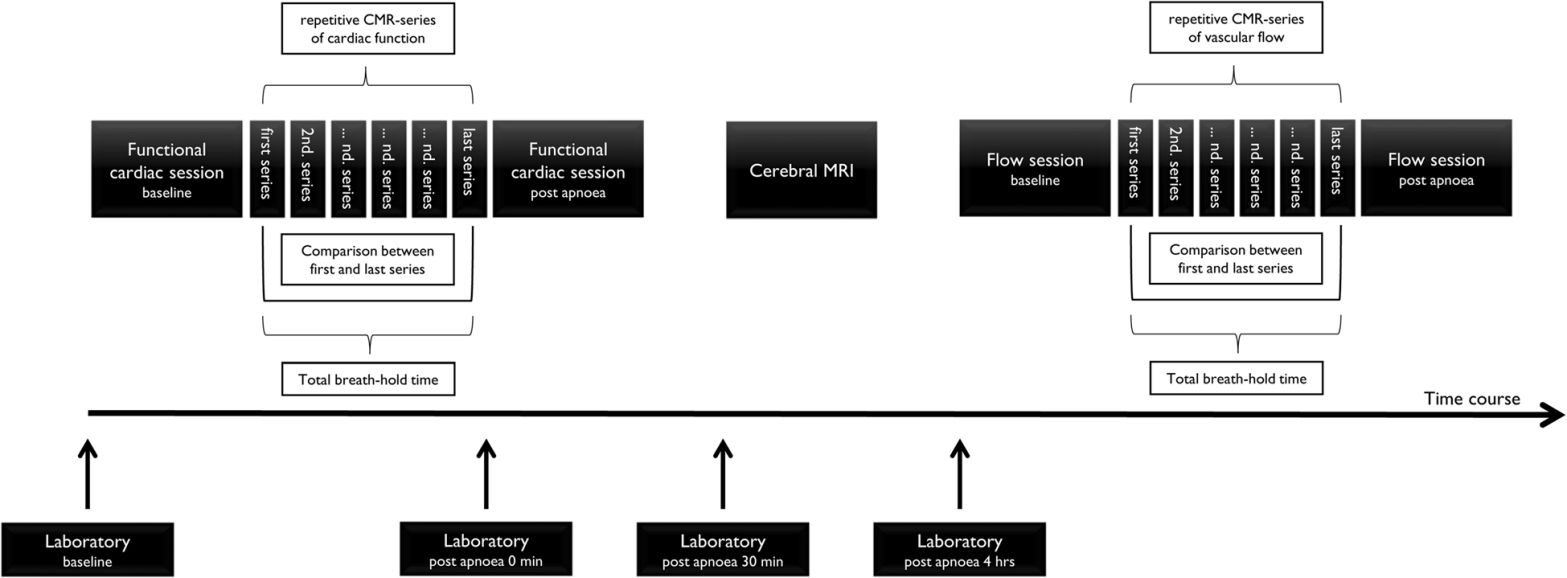
Illustration of the study protocol (CMR: cardiovascular magnetic resonance)

This “individual” approach close to personal best breath-hold amplifies the redistribution effects and supposedly exhibits the maximum effect on the cardiovascular system. Additionally, a brain MRI was performed at least 30 minutes after the first of the two breath-hold sessions to detect acute brain ischemia. Cardiac biomarkers and catecholamines were evaluated to detect cardiac damage (for time points see Fig. 1).

### Magnetic resonance imaging technique

All CMR studies were performed during voluntary breath-hold in maximal inspiration and in supine position using a 1.5 Tesla whole body scanner (Ingenia, Philips Healthcare, Best, The Netherlands). The protocol for the “functional cardiac session” consisted of retrospectively gated balanced steady state free precision cine imaging with 30 cardiac phases per slice. To assess functional changes under apnoea, three short axis (apical, midventricular, basal) cines as well as a vertical long axis cine  were acquired repeatedly over the course of apnoea. T2-mapping – indicative of myocardial oedema - using a gradient-spin-echo technique was performed in the same three slices in short axis orientation as the steady state free precision cine images prior and immediately after maximal apnoea [12].

MRI of the brain was performed using a 3 Tesla MRI scanner (Ingenia, Philips Healthcare) using a dedicated head coil. The protocol comprised a transverse T2-weighted turbo spin echo (TSE), a transverse fluid attenuated inversion recovery (FLAIR), a transverse T2*, a sagittal T1-weighted 3D gradient echo, and a transverse as well as a coronal diffusion weighted imaging (DWI) sequence.

The second CMR session was performed at least 4 h after the first “functional” cardiac session. This session was focused on flow measurements in the ascending and descending aorta, the pulmonary trunk, and both common carotid arteries using common 2D phase contrast imaging.

### Image analysis

Two radiologists blinded to the study protocol independently evaluated all images. The first (apnoea_early_) and last completed imaging data set (apnoea_late_) of each maximum apnoea were compared. Global cardiac function at resting conditions (left ventricular end-diastolic volume (LVEDV), end-systolic volume (LVESV), stroke volume (LVSV), and ejection fraction (LVEF) were determined from the short axis (ViewForum, Philips Healthcare) and normalized to the body surface area (BSA) using Mosteller’s formula [13]. LVEDV and LVESV were quantified manually by tracing the endocardial borders in all short axis slices. For functional parameters under apnoea LV volumes were determined using the modified Simpson rule. For the assessment of regional cardiac function, fractional shortening (FS) was assessed as described previously [14]. In short, the endocardial distance from the free LV lateral wall to the septal wall was measured in end-diastole (EDD) and end-systole (ESD) in apical, midventricular and basal slices in short axis orientation. FS was then calculated as followed: $FS=\frac{EDD- ESD}{EDD}\times 100$. For flow quantification, phase contrast imaging was analysed using dedicated software (ViewForum, Philips Healthcare). Borders of the ascending and the descending aorta, the pulmonary trunk and both common carotid arteries (CCA) were manually traced in the magnitude images and the region of interest was automatically copied to phase images with manual correction performed when deemed necessary. Maximum velocity (V_max_), mean velocity (V_mean_), mean flow (Q_mean_) and absolute stroke volume (SV) was assessed, respectively. T2 relaxation times were extracted from the T2 maps that were generated by using dedicated software (Intellispace Portal 9.0, Philips Healthcare). A circular region of interest (ROI) was then manually placed in the septal and lateral LV wall and averaged.

Cerebral MRI was analysed by the same two radiologists for focal diffusion restrictions as a sign of acute cerebral ischemia, cerebral micro-bleedings, and incidental findings. Peripheral oxygen saturation (SpO_2_) and heart rate (HR) were measured continuously during both CMR sessions using a CMR-compatible device (Expression MR400, Invivo, Gainesville, Florida, USA).

### Estimation of myocardial oxygen demand

Myocardial oxygen demand was estimated using the modified pressure work index as previously described (PWI mod = modified pressure work load index, P_systolic_ = systolic blood pressure, P_diastolic_ = diastolic blood pressure, HR = heart rate, CO = cardiac output, BSA = body surface area) [15]:

$$ PWImod=0.02+\left({P}_{systolic}\times HR\times 8.37\times {10}^{-4}\right)+\left(0.8\times {P}_{systolic}+0.2\times {P}_{diasystolic}\right)\times \frac{CO}{BSA}\times 8\times {10}^{-5}\Big) $$

### Laboratory testing

Urine was collected for baseline measurements 30 min before the first apnoea session started and 4 h thereafter, but before starting the second CMR session. Catecholamine levels, N-terminal pro-hormone of brain natriuretic peptide (NT pro-BNP), brain natriuretic peptide (BNP) and high sensitive troponin (hs-cT) were analysed from venous blood samples taken before, immediately after, 30 min, and 4 h after the first apnoea. All results (urine and blood samples) therefore reflect the effect of the first single breath-hold, but not those of repetitive apnoeal stages.

### Laboratory analyses

NT-pro BNP measurements were performed immediately after blood collection under routine conditions with the LOCI™-based NT-proBNP assay for Dimension™ VISTA 1500 (Siemens Healthcare Diagnostics, Eschborn, Germany). For BNP, hs-cT, and catecholamine analysis, aliquots were stored at − 80 °C. BNP and hs-cT were measured using commercially available, specific immunoassays (BNP and STA High Sensitive Troponin-I assay for ARCHITECT™, both Abbott Diagnostics, Wiesbaden, Germany). Plasma catecholamine levels were analyzed using a catecholamine reagent kit (Chromsystems Instruments & Chemicals GmbH; ord. no. 5000; Graefelfing, Germany) with a HPL Chromatography (Waters Corporation, Milford, Massachusetts, USA). Urine catecholamines were analysed by Bio Rad HPLC Agilent 1100 Series (Agilent Technologies, Waldbronn, Germany).

### Statistical analysis

Data are presented as mean +/- standard deviation (SD). Statistical analysis was performed using GraphPad Prism (version 7.02 for Windows, GraphPad Software, La Jolla, California, USA) and SAS version 9.4 (SAS Institut Inc., Cary, North Carolina, USA). Descriptive statistics are summarised as means and standard deviation (± SD). All parameters were compared using paired t-testing. Correlations were calculated using the Spearman’s rank correlation analysis. Statistical significance was defined as *p* < 0.05.

## Results

Seventeen elite apnoea divers (15 men) age of 40 ± 11 years were investigated under maximum breath-hold (height 183 ± 8 cm, weight 82 ± 11 kg, BSA 2.0 ± 0.17 kg / m^2^ and body mass index (BMI) 24.4 ± 2.3 kg /m^2^). Five of 17 divers regularly participate in various national and international competitions. The training frequency was 2.2 ± 1.6 training sessions a week. Training content and focus varied inter-individually. The breath-hold experience of all athletes was 4.5 ± 2.6 years. The personal breath-hold records were 5:20 ± 0:49 min. No comorbidities were found in any diver. Five divers have had a hypoxic blackout in their history.

Maximal breath-hold time in the “functional cardiac session” was 413 s and maximal breath-hold time in the “flow session” was 483 s. Mean time of breath-hold in the “functional cardiac session” was 297 ± 52 s and 276 ± 80 s in the “flow session” (*p* = 0.14). SpO_2_ levels gradually decreased from 99 ± 1% to 74 ± 14% (*p* < 0.001) in the “functional cardiac session” and from 99 ± 1% to 77 ± 15% (*p* < 0.001) in the “flow session”. No hypoxic loss of consciousness was observed. Physiological data of each participant are listed in Table 1.

**Table 1:** Demographical and physical characteristics of all apnoea divers

Participants	Age [years]	Sex	Height [cm]	Weight [kg]	Time of 1st BH [s] cardiac session	Baseline SpO_2_ [%] cardiac session	Minimal SpO_2_ [%] cardiac session	Time of 2nd BH [s] flow session	Baseline SpO_2_ [%] flow session	Minimal SpO_2_ [%] flow session
1	58	male	175	80	200	99	89	194	99	87
2	23	male	185	73	255	100	92	274	99	86
3	36	male	183	79	233	99	76	272	99	39
4	51	male	193	86	318	99	68	298	98	52
5	35	male	186	70	293	99	73	262	98	79
6	53	female	166	76	268	99	52	162	99	89
7	46	male	180	73	348	98	82	302	99	87
8	38	female	176	67	310	98	66	299	99	72
9	30	male	178	75	413	99	55	377	99	56
10	24	male	200	110	278	99	89	267	99	89
11	35	male	176	70	328	99	89	237	98	91
12	29	male	186	86	366	99	71	483	97	76
13	46	male	183	91	295	99	81	264	99	87
14	31	male	185	85	263	98	79	249	100	86
15	50	male	189	86	278	97	75	254	99	82
16	56	male	190	89	362	99	44	377	99	58
17	36	male	185	98	249	100	85	132	99	91
Ø	40		183	82	297	99	74	277	99	77
SD	11		8	11	53	1	14	80	1	15

*BH* breath-hold, *SpO*_*2*_ peripheral oxygen saturation

### Cardiac functional analysis

**Fig. 2:**
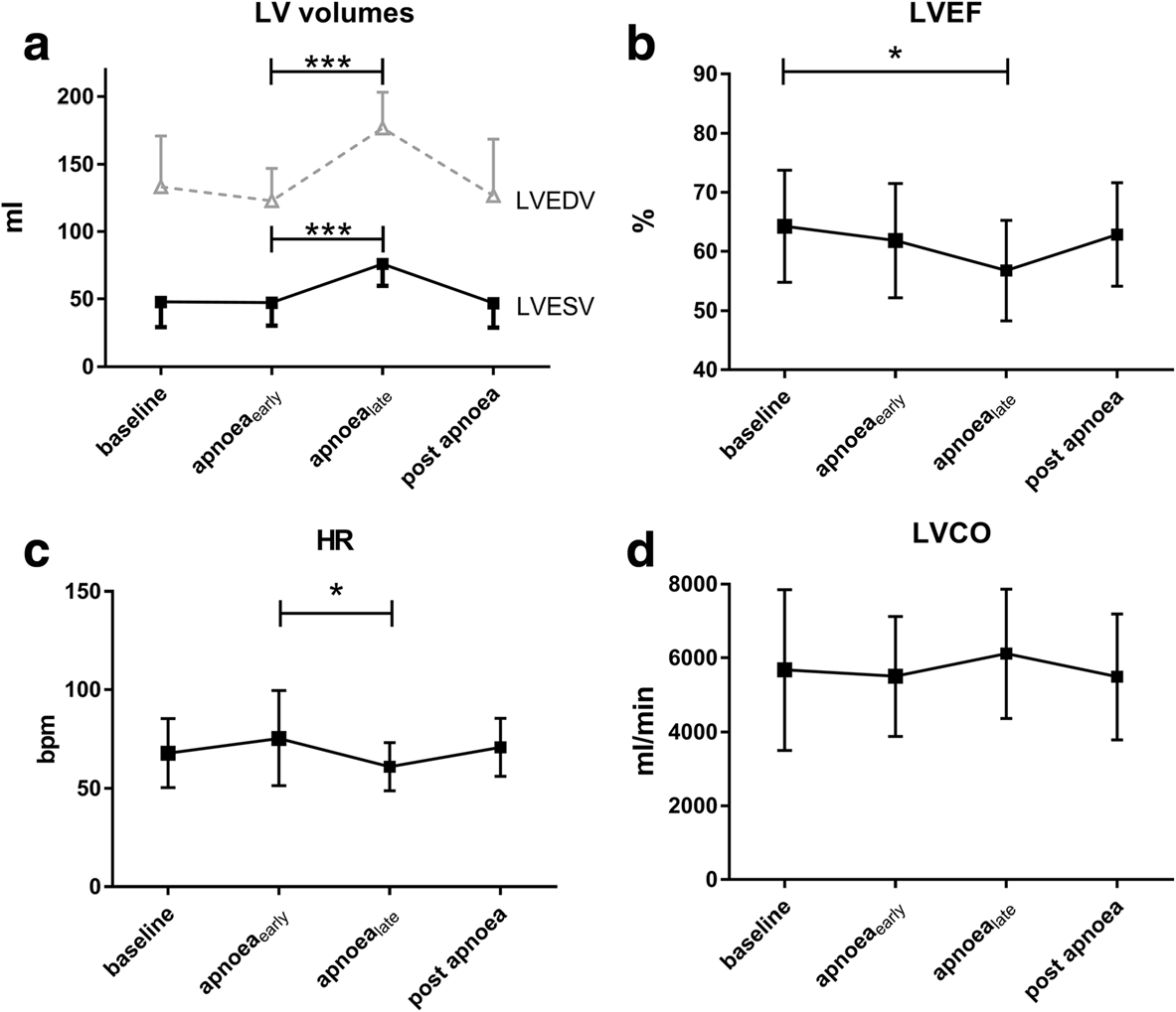
Left ventricular changes during apnoea. **a**) LV volumes: left ventricular volumes. **b**) LVEF: left ventricular ejection fraction, **c**) HR: heart rate, **d**) LVCO: left ventricular cardiac output (*p = < 0.05; **p = < 0.01; ***p = < 0.001; ****p = < 0.0001). Values are expressed as mean ± standard deviation

**Fig. 3:**
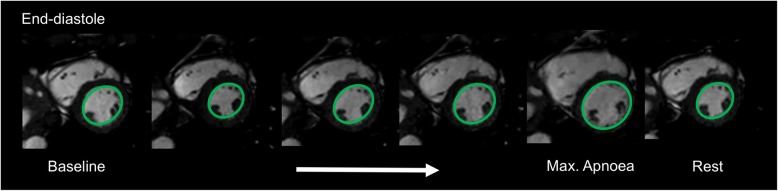
Representative image showing a progressive LV dilation over the course of apnoea in diastolic heart phase (subject 12)

In all participants, LVEDV, LVESV, and LVSV *increased* from onset to the end of apnoea (122.9 ± 24.2 ml vs. 176.9 ± 26.4 ml, *p* < 0.001; 47.3 ± 16.8 ml vs. 75.9 ± 16.3 ml, *p* < 0.001; 75.6 ± 16.9 ml vs. 101.0 ± 22.9 ml, *p* = 0.003) (Fig. 2a), while LVEF (Fig. 2b) *decreased* from 61.8 ± 9.4% to 56.8 ± 8.2% (*p* = 0.04). Despite a significant decrease of HR (75 ± 23 bpm vs. 61 ± 12 bpm; *p* = 0.028) (Fig. 2c), LV-CO remained unchanged (5.5 ± 1.6 l/min vs. 6.1 ± 1.7 l/min, *p* = 0.88) (Fig. 2d). A representative image series of gradual LV enlargement is shown in Fig. 3. FS decreased over the course of apnoea from 33.0 ± 6.0% to 23.8 ± 4.4% (*p* < 0.001), with the decrease in the apical slice (41.5 ± 7.4% vs. 27.9 ± 7.2%, *p* < 0.001) and the midventricular slice (30.7 ± 9.0% vs. 21.6 ± 4.4%, *p* < 0.001), whereas only a non-significant trend was observed in the basal slice (26.7 ± 7.1% vs. 22.6 ± 5.5%, *p* = 0.065) (see also Table 2).

**Table 2:** Parameters of CMR functional cardiac session

CMR functional cardiac session	Baseline ±SD before apnoea	Begin of apnoea ±SD apnoea_early_	End of apnoea ±SD apnoea_late_	Mean of difference at beginning vs. end of apnoea	SD of differences	95% confidence interval	*p*-value
HR [bpm]	67.9 ± 17.0	75.5 ± 23.3	61.1 ± 11.8	−14.8	24.4	−27.8 to −1.8	0.0282
LVEDV [ml]	133.4 ± 37.9	122.9 ± 24.2	176.9 ± 26.4	53.4	28.2	38.3 to 68.4	< 0.001
LVESV [ml]	48.0 ± 18.6	47.3 ± 16.8	75.9 ± 16.3	27.4	14.5	19.7 to 35.1	< 0.001
LVEF [%]	64.3 ± 9.2	61.8 ± 9.4	56.8 ± 8.2	−4.2	7.5	−8.2 to −0.2	0.0397
LVSV [ml]	86.4 ± 26.3	75.6 ± 16.9	95.1 ± 32.6	19.5	34.1	2.0 to 37.1	0.0014
LVCO [l/min]	5.7 ± 2.1	5.5 ± 1.6	6.1 ± 1.7	0.6	1.0	0.1 to 1.2	0.021
FS [%]							
apical	43.8 ± 5.9	41.5 ± 7.4	27.9 ± 7.2	−13.7	5.8	−16.7 to − 10.7	< 0.0001
midventricular	33.9 ± 7.1	30.7 ± 9.0	21.6 ± 4.4	−8.3	7.6	−12.4 to −4.3	0.0005
basal	30.1 ± 5.4	26.7 ± 7.1	22.6 ± 5.5	−3.2	6.0	−6.7 to 0.2	0.0646
mean	35.9 ± 4.8	33.0 ± 6.0	23.8 ± 4.4	−8.6	4.4	−10.9 to −6.2	<.0001

*HR* heart rate, *LVEDV* left ventricular end-diastolic volume, *LVESV* left ventricular end-systolic volume, *LVEF* left ventricular ejection fraction, *LVSV* left ventricular stroke volume, *LVCO* left ventricular cardiac output, *FS* fractional shortening, *SD* standard deviation; *p*-values for begin apnoea vs. end of apnoea

**Fig. 4:**
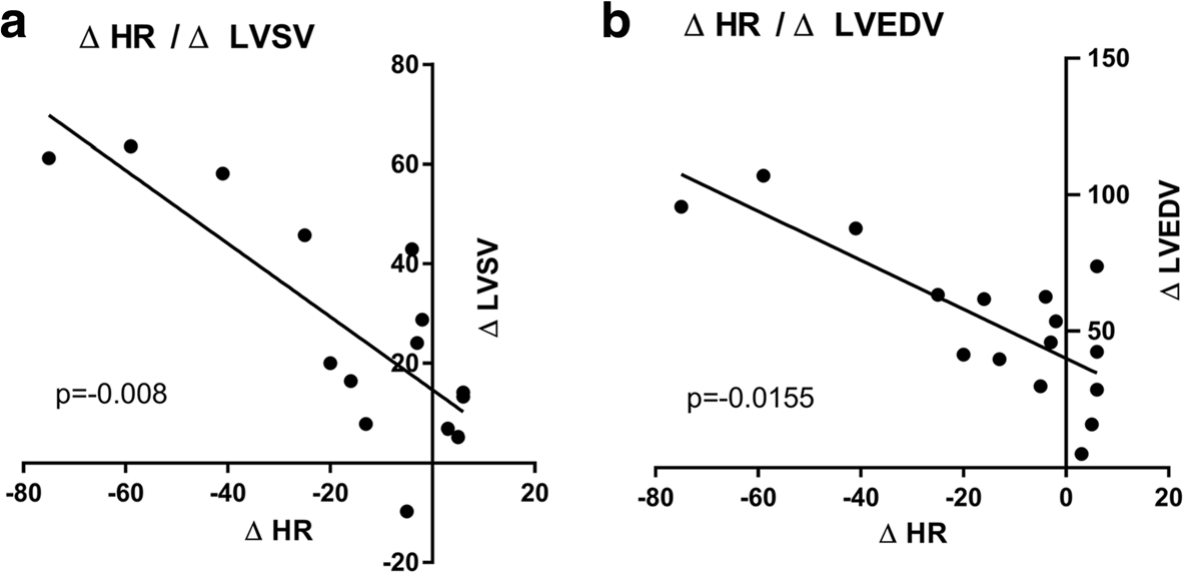
Correlation of **a**) ΔHR (Apnoea_early_ – Apnoea_late_) with ΔLVSV (Apnoea_early_ – Apnoea_late_, panel and **b**) ΔLVEDV (Apnoea_early_ – Apnoea_late_, panel respectively, using Spearman’s rank correlation (ΔHR with ΔLVSV: − 0.637, *p* = 0.008; ΔHR with ΔLVEDV: -0.592923; *p* = 0.0155). HR: heart rate, LVSV: left ventricular stroke volume; LVEDV: left ventricular end-diastolic volume

Changes in HR (∆HR) over the course of apnoea (Fig. 4) showed a significant negative correlation with the changes of LVSV (∆LVSV) (Spearman’s rank correlation analysis; correlation coefficient − 0.64, *p* = 0.008). Additionally, ∆HR over the course of apnoea had a significant negative correlation with the change of LVEDV (∆LVEDV) (Spearman’s rank correlation analysis; − 0.59; *p* = 0.016). This was less prominent when ∆LVEDV was normalized to BSA (Spearman’s rank correlation analysis; − 0.55; *p* = 0.028). In contrast, there was only a weak correlation between ∆HR and an increase in ∆LVESV (− 0,319; *p* = 0.23).

### Quantitative flow analysis

**Fig. 5:**
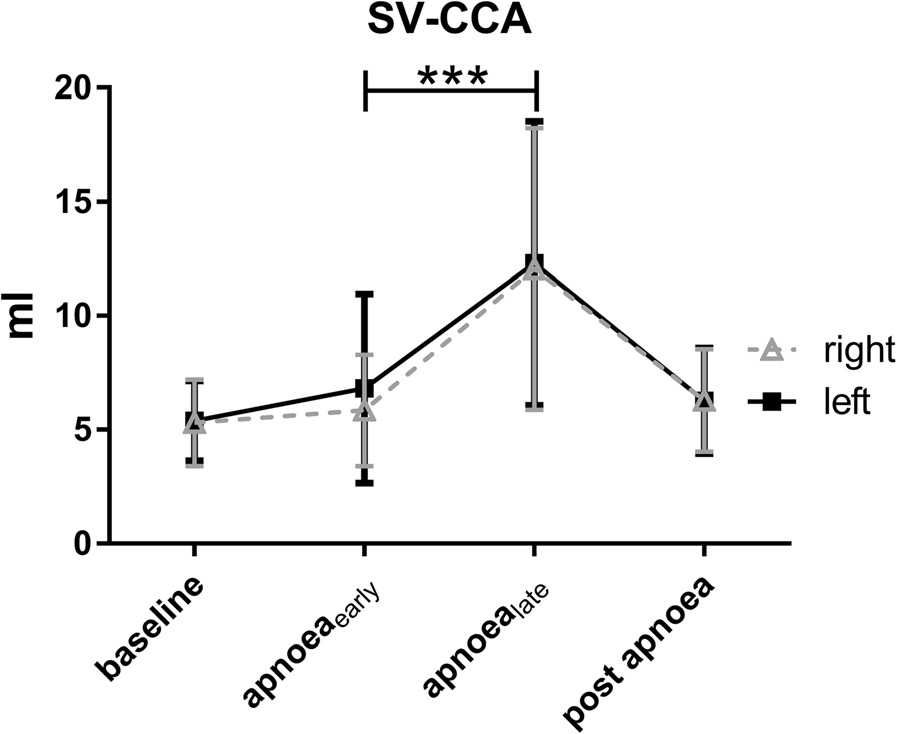
Stroke volumes of common carotid arteries during course ao apnoea. SV-CCA: stroke volume in common carotid arteries. Values are expressed as mean ± SD

While SV, V_max_, V_mean_, and Q_mean_ increased significantly in the ascending aorta and the pulmonary trunk during apnoea, no changes were observed in the descending aorta, indicating a preferential blood flow distribution to the heart and the brain. In addition, both CCAs showed a significant increase in SV (see Fig. 5), V_mean_ and Q_mean_ over the course of apnoea. All flow measurements are summarised in Table 3. A relevant shunt was neither observed at rest nor under apnoea (Qp/Qs: 1.06 ± 0.25 vs. 1.06 ± 0.19; *p* = 0.97).

**Table 3:** Parameters of CMR flow session

Vessel	Parameter	Baseline	Beginning of apnoea	End of apnoea	Mean Difference	SD of mean differences	95% confidence interval	*p*-value
Ascending aorta	stroke volume [ml]	72.3 ± 17.4	63.9 ± 21.3	96.3 ± 26.8	32.2	27.7	17.5 to 47.0	0.0003
	Q_mean_ [ml/s]	82.9 ± 27.8	74.3 ± 25.9	101.3 ± 31.3	29.5	32.2	12.3 to 46.7	0.0023
Descending aorta	stroke volume [ml]	40.8 ± 15.6	35.7 ± 16.0	41.4 ± 15.2	6.0	20.4	−5.3 to 17.2	0.2757
	Q_mean_ [ml/s]	45.0 ± 16.3	38.2 ± 10.6	43.4 ± 15.6	1.9	18.7	−9.4 to 13.2	0.7173
Pulmonary trunk	stroke volume [ml]	71.6 ± 31.1	64.6 ± 31.1	87.3 ± 31.8	38.1	40.5	10. 9 to 65.3	0.0109
	Q_mean_ [ml/s]	76.9 ± 26.5	63.5 ± 15.6	95.6 ± 28.4	45.3	32.6	21.9 to 68.6	0.0018
Right CCA	stroke volume [ml]	5.3 ± 1.8	8.9 ± 2.4	12.1 ± 5.9	6.9	5.5	3.3 to 10.4	0.0013
	Q_mean_ [ml/s]	6.1 ± 1.4	7.8 ± 2.8	11.2 ± 3.5	3.4	3.1	−5.0 to −1.8	0.0005
Left CCA	stroke volume [ml]	5.4 ± 1.7	6.8 ± 4.0	12.3 ± 6.0	6.9	5.6	−8.0 to −2.1	0.0022
	Q_mean_ [ml/s]	6.2 ± 1.3	7.7 ± 2.3	11.7 ± 4.0	4.0	3.8	6.0 to 2.0	0.0007

*CCA* common carotid artery; *p*-values for begin apnoea vs. end of apnoea, *SD* standard deviation, *Q*_*mean*_ mean flow

### Calculation of myocardial oxygen demand and oxygen supply

We found a decrease in HR (76 ± 23 bpm vs. 61 ± 12 bpm) and an increase in LVSV (75.6 ± 16.9 ml vs. 95.1 ± 32.6 ml) in this study. Using a previously reported increase of systolic and diastolic blood pressure from 135 ± 13 mmHg to 185 ± 25 mmHg [16], the estimated myocardial oxygen demand using the modified pressure work index [15] increases from 8.51 ml/min/100 g to 9.48 ml/min/100 g (increase of 11%) during apnoea.

### T2 mapping

Compared to baseline values, T2 relaxation times showed no significant change (51.7 ± 2.4 ms vs. 52.6 ± 2.5 ms, *p* > 0.05). 

### Laboratory analysis of catecholamine levels, NT pro-BNP, hs-cT, BNP

**Fig. 6:**
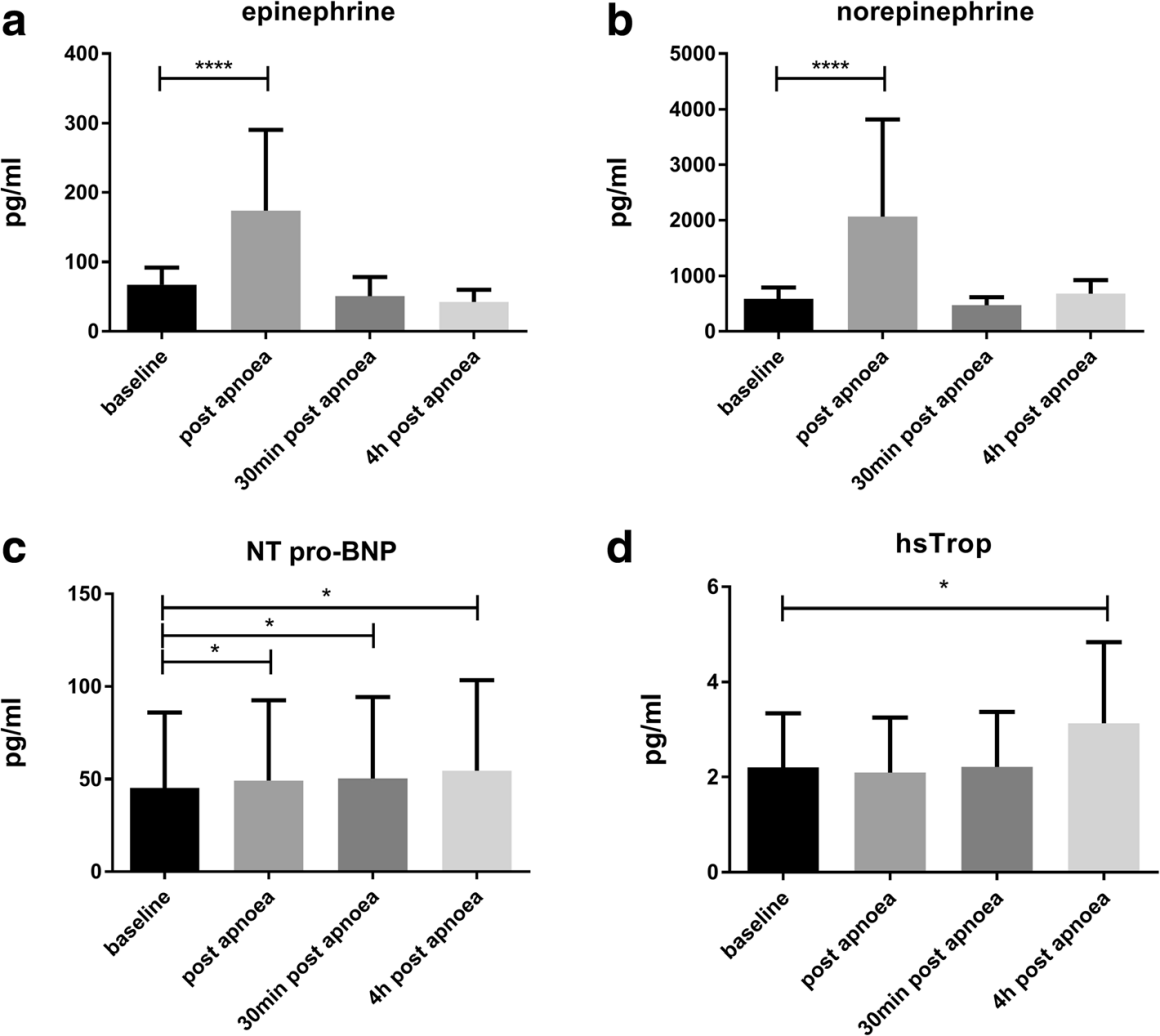
Serum parameters of **a**) epinephrine, **b**) norepinephrine, **c**) NT pro-BNP and **d**) high sensitive Troponin (hsTrop) under apnoea

Serum catecholamine levels (see Fig. 6a, b) showed a fast, increase immediately after apnoea onset (epinephrine from 67.5 ± 23.9 pg/ml to 173.8 ± 113.2 pg/ml (*p* < 0.001); norepinephrine from 590 ± 197 pg/ml to 2063 ± 1703 pg/ml (*p* < 0.001)). Serum catecholamine levels returned to baseline values as early as 30 min post apnoea (epinephrine: 50.9 ± 26.6 pg/ml; norepinephrine: 474.5 ± 138.1 pg/ml). Catecholamine levels derived from urine samples still showed a slight, but significant increase 4 h after apnoea compared to baseline conditions (epinephrine from 6.1 ± 2.0 pg/ml to 11.3 ± 6.1 pg/ml, *p* = 0.003; norepinephrine from 25.0 ± 20.1 pg/ml to 42.3 ± 22.6 pg/ml, *p* = 0.011).

NT pro-BNP increased slightly from baseline levels 45.9 ± 40.3 pg/ml to 49.3 ± 43.3 pg/ml immediately after apnoea (*p* = 0.011) and to 53.8 ± 49.4 pg/ml (*p* = 0.037) 4 h after breath-hold (see Fig. 6c). BNP could not be quantified in 7 out of 17 subjects due to values lower than the detection limit (< 10 pg/ml). Overall, there were no significant changes of BNP serum levels at any time point.

Hs-cT increased from baseline until 4 h after apnoea (2.2 ± 1.1 pg/ml vs. 3.1 ± 1.7 pg/ml, *p* = 0.026) (Fig. 6d). Compared to baseline levels, this resulted in a mean relative Hs-cT increase of 56%, but was still far from any pathological range.

### Cerebral MRI

DWI revealed neither acute nor sub-acute signs of cerebral ischemia. In one participant, a clinically irrelevant singular micro-bleeding formation located in the brain stem was observed. In two participants, unilateral fluid collections of the mastoid were observed and reported. No further incidental findings were observed.

## Discussion

In this present study, a holistic approach with state-of-the-art cardiac function evaluation, tissue characterization and biomarker analysis was performed to evaluate myocardial function, thoracic and supra-aortic blood flow, and their changes during maximal individual apnoea. The major findings of our study are a stepwise (1) increase of LVEDV, LVESV, LVSV and an unchanged CO, (2) decrease of LVEF and FS at the end of apnoea, (3) increase of supra-aortic blood flow without concurring flow changes in the descending aorta, and (4) an elevated hs-cT and NT-pro-BNP levels.

### Cardiac function

In the present study we were able to demonstrate a significant LV dilatation along with an increased LVSV, which is in line with a previous study [17], where increased EDD and ESD, an increase in SV and CO and a reduction in contractile function after an apnoea time of 3.7 ± 0.3 min was reported. In contrast to our results, neither bradycardia nor increased calculated systemic vascular resistance were observed [17], although both effects are part of the accepted concept of the diving response [8]. In a more recent study by Batinic et al., cardiac parameters (i.e. HR, LV volumes, LVEF, LVCO) taken at two time points of apnoea (minute 1 and minute 3) were compared [18]. These investigators found a significant increase in LVEDV and CO (112 ± 15 ml to 125 ± 15 ml; 5.4 ± 1.9 l/min to 6.0 ± 1.2 l/min), which was similar to our results (123 ± 24 ml to 177 ± 26 ml; 5.5 ± 1.6 l/min to 6.1 ± 1.7 l/min). In contrast to the previous results from Pingitore et al. [17] and the results of the present study, no changes in SV were observed (69 ± 12 ml to 69 ± 8 ml), while HR increased from 80 ± 15 bmp to 87 ± 16 bmp during apnoea [18].

Since the mammalian diving response to maximal voluntary apnoea considerably varies depending on the examined individual and the study setup, the at first apparently contradictory results of the three studies might be explained by the breath-hold duration [5, 9, 19]. In contrast to previous studies focusing on physiological changes during apnoea, the breath-hold time in the present study was considerably longer (297 ± 99 s vs. 234 ± 66 s; 199 ± 11 s; 210 ± 70 s) [5, 9, 19]. However, even though individual responses may vary, it is known that physiological changes are most notable at the end of apnoea [9, 20, 21]. In this context it is important to mention that the previous studies [17, 18] used predefined time points for data collection, which will not necessarily coincide with the individual maximum breath-hold duration of each athlete. We have therefore decided to use a minimal breath-hold duration of 270 s to eliminate the possible shortcomings of a too short apnoea duration in the previous studies [5, 9, 19–21]. Therefore, one can speculate that the shorter breath-hold durations registered in both previous studies [10, 17] are not suitable to push all compensatory mechanisms to their limits, and that a predefined time point might lead to undersampling. This is further supported by the fact that SpO_2_ decreased more profoundly in our study compared to the study from Pingitore et al. (from 99 ± 1% to 74 ± 14% vs. 97 ± 0.2% to 84 ± 2%) [17].

We found a relative increase in LVSV of 30 ± 48% during apnoea, but a decrease in FS and LVEF. FS depends on inter-ventricular dimensions and is affected by ventricular filling. Ejection fraction, in contrast, is a relatively load independent surrogate parameter for cardiovascular performance. In general, the efficiency of myocardial performance is determined by preload, afterload and contractility [22, 23]. An increase in afterload will therefore result in decreased efficiency of myocardial performance. In case of prolonged breath-hold the peripheral chemoreflex regulation, the elevated sympathetic nerve activity and the increase in norepinephrine will lead to peripheral vasoconstriction and hypertension [5, 24] and subsequently to bradycardia via the baroreflex [25]. In accordance with this established physiological pathway, we observed a significant increase in norepinephrine levels to above the upper cut-off limit of > 420 pg/ml and a decrease in HR at the end of apnoea. Therefore, the HR decrease and the concommitant increase of both ventricles may be seen as an indirect visualization of the aforementioned baroreflex (Fig. 4).

### Biomarkers

NT-proBNP was elevated early after maximal apnoea. Although some authors describe BNP as an “emergency” cardiac hormone against ventricular overload [26], the observed elevations of pro-BNP were only minor and far from pathological levels. Nevertheless, in absence of other triggers even this small increase may be regarded as an indicator for LV wall stress. Although an increase in hs-cT was found in this study, the normal T2 relaxation times directly after apnoea may indicate that the increased hs-cT may be more attributable to the LV dilatation and not to acute and persistent myocardial damage. This may further be supported by the fact that elevated cardiac troponin (cT) levels are also commonly found in patients with dilated cardiomyopathy [27].

In addition, myocardial perfusion and oxygen consumption is dependant on various parameters. At the end of apnoea, HR decreases while SV and systolic and diastolic blood pressure increase. These physiological changes translate into an increase of estimated oxygen demand in our study from 8.5 ml/min/100 g to 9.5 ml/min/100 g (i.e. only by 11%). However, this increase in demand may be assumed to be outweighed by a theoretical increase of approximately 40% of coronary perfusion due to increase of the diastolic blood pressure. It is of note that these theoretical considerations are based on healthy subjects without any coronary morbidities.

### Clinical context

The human diving response (i.e. bradycardia, peripheral vasoconstriction, increased blood pressure) helps to preserve O_2_ in case of apnoea [28]. These protective mechanisms against hypoxia are triggered by apnoea per se and are augmented by face immersion [29]. The constriction of intramuscular and dermal vessels results in an increased total peripheral resistance and thus in an increased blood pressure [9, 30]. Due to peripheral vasoconstriction and reduced blood flow, the remaining circulating blood flow is redistributed to more hypoxia sensitive organs such as the brain [19, 25]. In the present study, only minimal blood flow changes were seen in the descending aorta while blood flow in the ascending aorta and the carotid arteries massively increased, indicating that even the gastrointestinal tract is excluded from blood flow redistribution in the case of hypoxia. This perfusion preference of the cerebrum emphasises the efficiency of the body’s compensatory mechanism to avoid hypoxic damage of the brain. Accordingly, cerebral MRI showed no case of acute ischemia-induced brain injury, indicating the effectiveness of the compensatory mechanisms, even in case of breath-holds longer than 8 min (Table 1, subject 12).

 Prolonged apnoea is not exclusively seen in breath-hold divers, also patients with obstructive sleep apnoea (OSA) show compensatory mechanisms to avoid brain damage [31]. Patients with OSA show an increase in cerebral blood flow [32, 33], elevated sympathetic activity [34], elevated arterial blood pressure [35], and an increase in norepinephrine levels [31]. Interestingly, LV and right ventricular (RV) afterload are increased and cardiac arrhythmia is commonly seen [36]. OSA is independently associated with coronary artery disease, atherosclerosis, hypertension, stroke, endothelial function and myocardial infarction [37, 38]. A main problem in understanding the underlying pathophysiology stems from the lack of an adequate clinical model to simulate OSA [39]. So far, hypoxic gas mixtures have been used to mimic hypoxia in humans [40], but because of the resulting hyperventilation, these models are more representative for high altitude environments than for OSA. In addition, the transmissibility of animal models is also limited. Apnoea divers are mostly free of comorbidities, and our study shows that even a short episode of hypoxia affects the cardiovascular system. Therefore, voluntary extended breath-hold might be taken as a clinical relevant model to simulate short term changes due to hypoxia [41], although the exposure levels to hypoxemia differ significantly [41]. In this context it should be noted that this study was performed with trained athletes, and that a transfer of these findings to patients with cardiovascular diseases and obstructive sleep apnoea should be done with caution.

Patent foramen ovale has been demonstrated to have a higher prevalence in patients with obstructive sleep aponoea compared to healthy controls, and is suspected to inrease nocturnal oxygen desaturation in these patients [42] and to enhance other pathologic conditions associated with OSA [43]. In both scuba and apnoea divers knowledge about the implications of a patent foramen ovale regarding incidence and severity of decompression sickness is scarce [44], especially because it is unkown if recurrent decompression sickness is a result of a patent foramen ovale, the inabiliy to adopt a more conservative diving style, or both [45]. In the present study, no relevant changes of Qp/Qs (the stroke volume in the ascending aorta relative to the stroke volume in the pulmonary trunk) and thus no indication for a cardiac shunt, was found.

Cardiac dysrhythmia or irregular heartbeats (mainly premature ventricular excitations) were observed in 14 of 17 divers at the end of apnoea and during the early recovery phase (example shown in Additional file 1: Figure S1). It is tempting to speculate that the massive LV and RV dilatation triggers cardiac depolarization and repolarization. However, ECG quality was limited in this study and did not allow for a comprehensive analysis.

### Limitations

Measurment accuracy (CMR and SpO_2_) might be limited at the end of apnoea due to e.g. motion artefacts (CMR), peripheral vasoconstriction (SpO_2_), and other technical restrictions. Blood pressure data is not available as invasive blood pressure measurement was not performed due to ethical considerations and automatic non-invasive blood pressure measurement failed due to the high and dynamic changes in blood pressure during apnoea. Due to the chosen CMR imaging protocol with only limited coverage of the RV, neither volumetric nor functional RV data are available. Future studies should also focus on effects of hypxoxia on pulmonary vasoconstriction and their effects on the RV function.

## Conclusion

Compensatory effects of prolonged apnoea, including a massive LV dilatation and an increase in norepinephrine levels, substantially burden the cardiovascular system. This hemodynamic cardiac stress results in increased hs-cT and NT-pro-BNP, and leads to a reduction of FS. CMR tissue characterisation did not reveal acute myocardial injury, indicating that the resulting cardiovascular stress does not exceed compensatory short-term physiological limits in healthy elite divers. However, these compensatory mechanisms could overly tax those limits in subjects with pre- existing cardiac disease. Also, repetitive apnoea over decades, rather than over years as observed in our study population, may reveal different findings and may have a different impact on the cardiovascular system. For divers interested in competitive apnoea diving, a comprehensive medical exam with a special focus on the cardiovascular system may be warranted.

## Additional file

Additional file 1: **Figure S1.** Screenshots of monitored arrhythmia in different subjects (a-d) and in early recovery phase (e). (JPG 161 kb)

## Abbreviations

BMI: Body mass index
BNP: Brain natriuretic peptide
BSA: Body surface area
CCA: Common carotid arteries
CMR: Cardiovascular magnetic resonance
CO: Cardiac output
cT: Cardiac troponin
DWI: Diffusion weighted imaging
EDD: End-diastolic dimension
EDV: End-diastolic volume
EF: Ejection fraction
ESD: End-systolic dimension
ESV: End-systolic volume
FLAIR: Fluid attenuated inversion recovery
FS: Fractional shortening
HR: Heart rate
hs-cT: High sensitive troponin
LV: Left ventricle/left ventricular
MRI: Magnetic resonance imaging
NT-pro-BNP: N-terminal pro-hormone of brain natriuretic peptide
OSA: Obstructive sleep apnoea
pO_2_: Partial pressure of O_2_
PWI: Pressure work index
Qmean: Mean flow
ROI: Region of interest
RV: Right ventricle/right ventricular
SD: Standard deviation
SpO_2_: Peripheral oxygen saturation
SV: Stroke volume
TSE: Turbo spin echo
V_max_: Maximum velocity
V_mean_: Mean velocity
