# Supplementary figures and images for: Cardiovascular magnetic resonance assessment of acute cardiovascular effects of voluntary apnoea in elite divers
Source: J Cardiovasc Magn Reson. 2018 Jun 18;20:40. doi: 10.1186/s12968-018-0455-x (PMC6004697; doi:10.1186/s12968-018-0455-x)

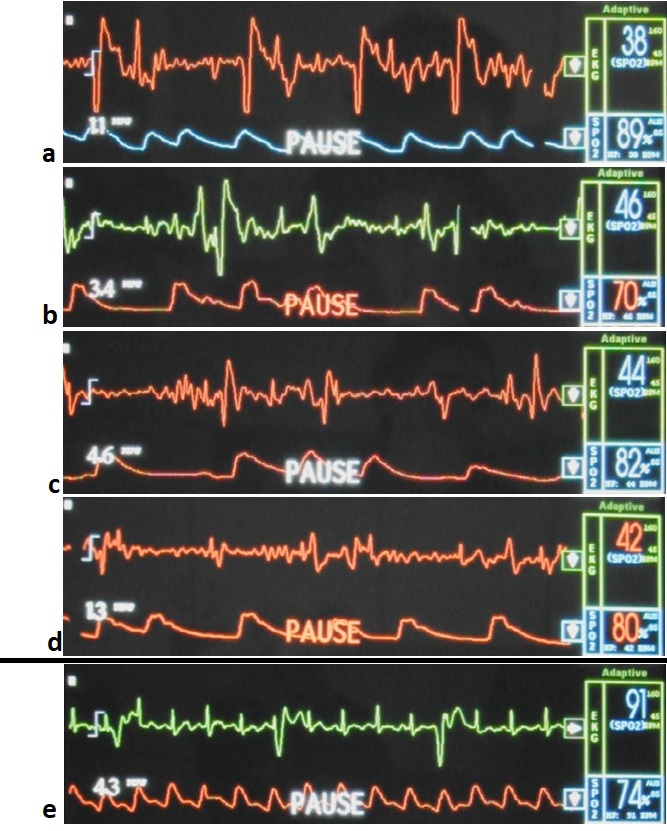

Supplement: Supplementary file 1 — Figure S1. Screenshots of monitored arrhythmia in different subjects (a-d) and in early recovery phase (e). (JPG 161 kb) [file 12968_2018_455_MOESM1_ESM.jpg]
